# Supplementary material for: Tailoring the Emission Behavior of WO3 Thin Films by Eu3+ Ions for Light-Emitting Applications
Source: Nanomaterials (Basel). 2022 Dec 20;13(1):7. doi: 10.3390/nano13010007 (PMC9824464; doi:10.3390/nano13010007)
Supplement: Supplementary file 1 [file nanomaterials-13-00007-s001.zip › nanomaterials-2037496-supplementary.pdf]

nanomaterials

Supplementary Materials

Tailoring the Emission Behavior of WO_3 Thin Films by Eu^{3+}Ions for Light-Emitting Applications

V. S. Kavitha [1,2], V. Biju [2], K. G. Gopchandran [1], R. Praveena [3], C. K. Jayasankar [4], Wanichaya Mekprasart [5], Kanokthip Boonyarattanakalin [5], Wisanu Pecharapa [5,*] and V. P. Mahadevan Pillai [1,6,*]

1 Department of Optoelectronics, University of Kerala, Kariavattom, Thiruvananthapuram 695581, India
2 Department of Nanoscience and Nanotechnology, University of Kerala, Kariavattom, Thiruvananthapuram 695581, India
3 Department of Physics, Gayatri Vidhya Parishad College of Engineering (A), Visakhapatnam 530048, India
4 Department of Physics, Sri Venkateswara University, Tirupati 517502, India
5 College of Materials Innovation and Technology, King Mongkut's Institute of Technology Ladkrabang, Bangkok 10520, Thailand
6 Amrita School of Physical Science, Amrita VishwaVidyapeetam, Amaravathi Campus, Coimbatore 641112, India
* Correspondence: wisanu.pe@kmitl.ac.th (W.P.); vpmpillai9@gmail.com (V.P.M.)

Supporting Information

$$D_{hkl} = \frac{k\lambda}{\beta_{hkl}\cos\theta_{hkl}} \qquad \text{Equation (S1)}$$

where k is a constant and its value is 0.9 for spherical shape particles. λ=1.5406 Å, is the wavelength of the X-ray radiation (CuKα line) used for recording the diffractograms. β_{hkl} stands for the full width at half maximum intensity (FWHM) of the intense (200) peak and θ_{hkl} denote half of the diffraction angle corresponds to intense (200) peak.

$$T\tan\theta_{hkl} = \frac{\lambda}{D_{hkl}\cos\theta_{hkl}} - \beta_{hkl} \qquad \text{Equation (S2)}$$

where T denotes the strain in the films.

$$\delta = \frac{1}{D^2} \qquad \text{-Equation (S3)}$$

where δ denotes the dislocation density.

$$\alpha(h\nu) = A(h\nu - E_g)^n \qquad \text{Equation (S4)}$$

where 'A' is a constant called the band edge constant and is associated with the band tailing. hν corresponds to the energy of the incident photon and 'n' determines the nature of electronic transition.
